# Author Correction: Miniscope3D: optimized single-shot miniature 3D fluorescence microscopy

**DOI:** 10.1038/s41377-023-01146-x

**Published:** 2023-04-17

**Authors:** Kyrollos Yanny, Nick Antipa, William Liberti, Sam Dehaeck, Kristina Monakhova, Fanglin Linda Liu, Konlin Shen, Ren Ng, Laura Waller

**Affiliations:** 1 UCB/UCSF Joint Graduate Program in Bioengineering, University of California, Berkeley, CA 94720 USA; 2 Department of Electrical Engineering & Computer Sciences, University of California, Berkeley, CA 94720 USA; 3 TIPs Department, Université libre de Bruxelles (ULB), 1050 Brussels, Belgium

**Keywords:** Microscopy, Imaging and sensing

Correction to: *Light: Science & Applications*

10.1038/s41377-020-00403-7 published online 02 October 2020

After publication of this article^1^, it was brought to our attention that the Reference 30 should be removed from the article, and all reference numbers after reference 30 should be adjusted accordingly and moved up a number.

Along with this change, the superscript citations in the text body are all correct except for: The sentence “Once the adaptive stitching mask is obtained, the writing instructions per block can be generated using TipSlicer^36,37^” in the section titled “Phase mask fabrication” should now only reference bibliography item number 36.

The original publication has been corrected.
