# Supplementary material for: Miniscope3D: optimized single-shot miniature 3D fluorescence microscopy
Source: Light Sci Appl. 2020 Oct 2;9:171. doi: 10.1038/s41377-020-00403-7 (PMC7532148; doi:10.1038/s41377-020-00403-7)
Supplement: Supplementary file 1 — Supplemental Material [file 41377_2020_403_MOESM1_ESM.pdf]

Miniscope3D: Optimized Single-shot Miniature 3D Fluorescence Microscopy: Supplementary Material

Kyrollos Yanny[1,*], Nick Antipa[2,*], William Liberti[2], Sam Dehaeck[3], Kristina Monakhova[2], Fanglin Linda Liu[2], Konlin Shen[1], Ren Ng[2], and Laura Waller[1,2]

[1] UCB/UCSF Joint Graduate Program in Bioengineering, University of California, Berkeley, CA, 94720, USA
[2] Department of Electrical Engineering & Computer Sciences, University of California, Berkeley, CA, 94720, USA
[3] TIPs Department, Université libre de Bruxelles (ULB), 1050 Brussels, Belgium
[*] Corresponding authors: kyrollosyanny@gmail.com & naantipa@gmail.com

This document provides supplementary information for "Miniscope3D: Optimized Single-shot Miniature 3D Fluorescence Microscopy". We provide details for the axial and lateral resolution of the system, the depth of focus, and the field-varying forward model analysis shown in the paper. In addition, we expand on our choice of reconstruction grid, regularization parameter, the advantages of using a microlens array over a diffuser, signal-to-noise ratio (SNR) comparison with other designed phase masks, the effect of sparsity on reconstruction quality, and the algorithm for adaptive stitching for two-photon polymerization 3D printers.

1. MICROLENSES VS GAUSSIAN DIFFUSER

For our phase mask, we choose a microlens array instead of the Gaussian diffuser used in our previous work[1]. This is because the microlenses can achieve point spread functions (PSFs) with higher SNR and frequency content than the diffuser (see Fig. S1), due to their better concentration of light in focus. Microlenses focus light into small focus spots, with dark areas between them, as opposed to the diffuser, which has some light spread between the caustics, generating unwanted low frequencies in the PSFs. Sharper focus spots in the microlens PSF mean that the SNR of the measurements is better and the inverse problem better posed. While using fewer focal spots would improve 2D measurement SNR and resolution, using a small number of microlenses does not provide enough multiplexing to gain 3D capability over a large depth range.

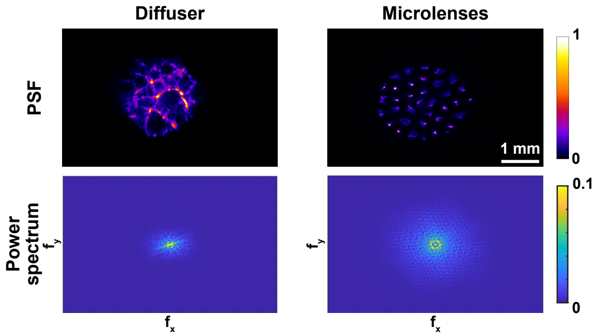

Fig. S1. Comparison of experimental PSFs resulting from a Gaussian diffuser and our microlens phase mask. The microlenses generate PSFs with more high-frequency content, as seen in the power spectrum. The microlenses also have better light concentration; to achieve the same brightness as the microlenses PSF, the diffuser requires 4× the exposure time.

2. AXIAL RESOLUTION

We determined the axial resolution by imaging a thin layer of 4.8 μm fluorescent beads. Because it is difficult to controllably place two beads at specific axial separation distances, raw data from a single bead at different depths are digitally added in order to synthesize a measurement of two layers of beads with varying separations. Figure S2 shows that we achieve a uniform 15μm axial resolution across our depth range of 360 μm. This closely matches with the axial full-width-half-maximum (FWHM) we observe in the 3D fluorescent beads sample in the main-paper *Results* section.

3. LATERAL RESOLUTION

Examining a single microlens, the Rayleigh criterion defines the minimum resolvable separation of two diffraction-limited spots on the sensor, $\delta x'$, in terms of the wavelength, λ, the microlens clear aperture, Δ_{ML}, and the distance from the mask to

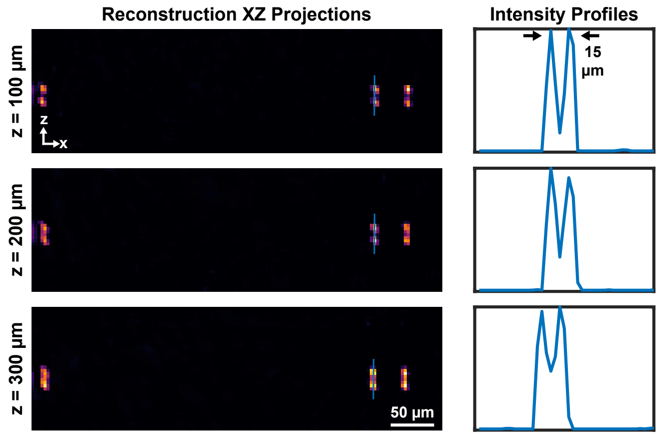

Fig. S2. Reconstructions results demonstrating 15μm axial resolution across our depth range. On left are x-z projections of the 3D reconstruction for the case of two layers of 3 beads each, separated by 15 μm axially. At right we show cross-cuts of the projections demonstrating clear resolving of the beads. The rows show results for placing the pairs of beads at different axial distances from the native focus plane.

the sensor, t:

$$\delta x' = \frac{1.22\lambda t}{\Delta_{ML}} = M\delta x \quad \text{(S1)}$$

Here we have used the fact that two points in object space separated by δ_x will appear as a separation of $M\delta_x$ on the sensor. Thus, we need to calculate the magnification of our system.

We use ray transfer matrices (with a paraxial approximation) to evaluate the magnification of the system. The system ABCD matrix is:

$$\begin{bmatrix} A & B \\ C & D \end{bmatrix} = \begin{bmatrix} 1 & t \\ 0 & 1 \end{bmatrix} \begin{bmatrix} 1 & 0 \\ -1/f_\mu & 1 \end{bmatrix} \begin{bmatrix} A_G & B_G \\ C_G & D_G \end{bmatrix} \begin{bmatrix} 1 & Q \\ 0 & 1 \end{bmatrix} \quad \text{(S2)}$$

and the system magnification, which is used in the lateral resolution derivation, is:

$$M = A = \left(1 - \frac{t}{f_\mu}\right) A_G + tC_G \quad \text{(S3)}$$

where A_G, B_G, C_G, & D_G are elements for the GRIN's ray transfer matrix ($A_G = 0.0725$, $B_G = 1.6931$, $C_G = -0.599$, and $D_G = 0.124$) and t is the distance from the phase mask to the sensor. Given that f_μ, the microlens focal length, ranges from 7 mm to 25 mm, combined with the small value for A_G, this results in the first term, $(1 - t/f_\mu)A_G$, being negligible and the magnification can be approximated simply as tC_G. This shows that for our system, the magnification is given by:

$$M \approx tC_G \quad \text{(S4)}$$

Substituting Eq. S4 into Eq. S1 and solving for Δ_{ML}, we get an expression for the microlens clear aperture needed for a target object resolution:

$$\Delta_{ML} = \frac{1.22\lambda t}{M\delta x} \approx \frac{1.22\lambda}{C_G \delta x} \quad \text{(S5)}$$

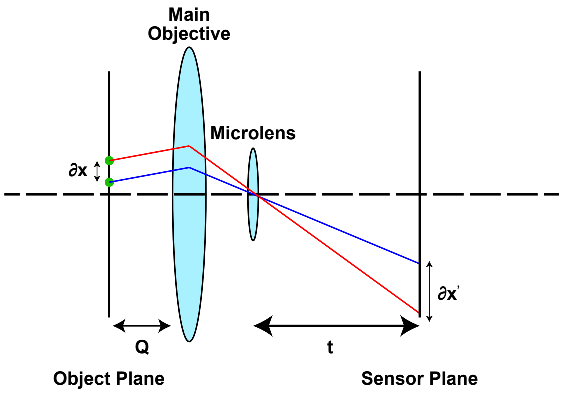

Fig. S3. Lateral resolution derivation. Examining a single microlens placed immediately after the main objective.

4. DEPTH OF FOCUS

We aim to determine the microlens depth-of-focus (DoF), defined as the distance that a point source in-focus can move axially before the blur spot on the camera sensor is bigger than a target circle of confusion radius, γ_c. To do so, we examine a single microlens' image in the GRIN entrance pupil for an object at distance z from the first principal plane of the GRIN. As the object moves axially by a distance d_{ML}, we can use similar triangles to derive (see Fig. S4 for variable definitions):

$$\frac{y}{d_{ML}} = \frac{\Delta_{EP}}{d_{ML} + z + L} \approx \frac{\Delta_{EP}}{L} \quad \text{(S6)}$$

where Δ_{EP} is the radius of the microlens' clear aperture in the entrance pupil (i.e. object side) of the GRIN and L is the distance from the first principal plane to the entrance pupil. Given that $L = 13\ mm$ is much larger than z, d_{ML}, which are on the order of 0.2 mm, we drop both z and d_{ML}. By substituting $y = \gamma_c/M$ into Eq. S6, we can solve for the microlens DoF as a function of our system parameters:

$$d_{ML} = \frac{\gamma_c L}{\Delta_{EP} M} \quad \text{(S7)}$$

Since the entrance pupil of the GRIN is very far from the object (it is approximately telecentric in object space), the object axial position is negligible in determining the microlens DoF. Designing for $\gamma_c = 12\ \mu m$, a circle-of-confusion smaller than the diffraction-limited spot size, $|M|\delta_x$, and using $\Delta_{EP} = 4\ mm$ (calculated using Zemax for a microlens with a clear aperture of 300 μm), we determine the DoF to be $\pm 20\ \mu m$.

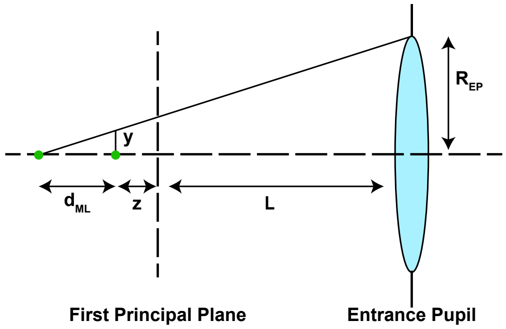

Fig. S4. Depth-of-focus (DoF) derivation setup, with distance variables defined.

5. CHOICE OF RECONSTRUCTION GRID

To successfully reconstruct **v**, we should define the reconstruction grid with sufficient sampling to realize the best resolution possible, but without oversampling, which increases computation and memory requirements. The theory above defines a band-limit for the measurements, so our goal is to use a sensor with a matching effective pixel size. In our architecture, increasing the sensor pixel size directly corresponds to increased lateral reconstruction voxel size and lower final resolution. Because of complicated interactions between nonlinear reconstructions and grid size, we determine our choice of lateral sampling empirically by binning the raw data from the resolution tests in the main paper *Results* section by $2\times$, $4\times$, and $8\times$ and evaluating the final resolution. We find that the resolution begins to degrade between $4\times$ and $8\times$ binning, so we operate at $4\times$ binning. This results in our sensor's effective object-space pixel size being 1.7 μm, which is sufficiently below the 2.76 μm minimum feature size that we observe experimentally. Note that the ability to use on-chip binning allows our approach to read data faster than a conventional LFM, which cannot use conventional on-chip binning without resolution loss. This allows us to achieve a 40 volume-per-second measurement rate using a low-cost USB 2.0 camera.

The choice of axial sampling informs our sampling interval during calibration (main-paper *Calibration* subsection). We measure every 5 μm, and perform axial binning (summing of consecutive PSFs) at $1\times$, $2\times$, and $4\times$. We find $1\times$ yields the best results. The resulting 5 μm axial sampling is reasonable given the empirically observed 15 μm axial resolution. Hence our choice of grid balances fast frame rates and efficient reconstruction with image quality and resolution.

6. CHOICE OF REGULARIZATION PARAMETER

One important parameter in our optimization problem is the regularization parameter τ. The regularization parameter sets the trade-off between the data fidelity term and our sparsity prior. In practice, this parameter sets the balance between preserving image details and noise reduction. Very small values of τ will preserve sharp details in our object; however, the reconstructions can be noisy. Very large values will suppress noise, but also suppress the object's details with it.

To test the reconstruction quality as a function of the regularization parameter, we ran our 3D reconstruction algorithm on the experimental resolution target data at $z = 270$ μm with values of τ ranging from 10^{-14} to 10^{-1}. Figure S5(a) shows that the reconstructions and the data fidelity term are stable for a wide range of τ values. As expected, for very large values of τ, the Total Variation (TV) prior over-regularizes the image, resulting in smoothed out details.

Since the experimental data lacks ground truth to compare against, we simulate a raw measurement by running our 3D shift-varying forward model on a two-photon microscopy zebra fish 3D dataset with our measured PSFs and adding realistic additive white Gaussian noise. The measurement is then processed with values of τ ranging from 10^{-14} to 10^{-1}. Figure S5(b) shows a trend similar to experimental results - the mean-squared error is stable for a large range of τ values, with over-smoothed reconstructions as τ gets very large. We note that all the data shown in the main paper was processed using the same value of τ, which further show that once a good value for τ is found, it can be used to process different classes of objects. While it may be possible to fine-tune τ for each measurement to achieve better performance, it is, however, more practical for users to use the default value. If the user is to fine-tune τ, we recommend using the largest value of τ that still preserves the object's fine details.

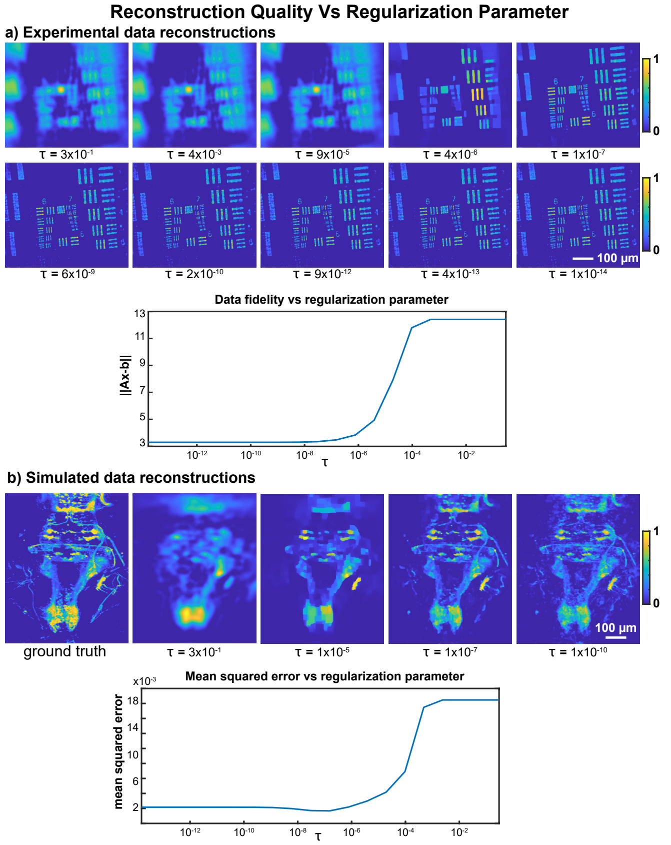

Fig. S5. Reconstruction quality as a function of regularization parameter, τ. (a) Maximum intensity projections of an experimental volume reconstructed with different τ settings, along with a plot of the data fidelity term as a function of τ on a semi-log scale. (b) Maximum intensity projections of a simulated volume reconstructed with different τ settings, along with a plot of mean-squared error as a function of τ on a semi-log scale. The results demonstrate the stability of reconstructions for a large range of τ values.

7. 2D MINISCOPE PSNR COMPARISON

Our Miniscope3D design is aimed at 3D imaging, but because it is smaller and lighter weight than 2D Miniscope, it might be useful in applications that only require 2D imaging. Because of the inherent aberrations in the GRIN lens, the 2D Miniscope does not achieve its full-aperture diffraction-limited resolution and our Miniscope3D resolution is only marginally worse than the 2D. However, we do suffer from reduced SNR as compared to the 2D Miniscope, because our PSFs spread the light over a larger area than a focused 2D Miniscope. To quantify this loss of SNR, we simulate measurements using on-axis PSFs from both our device and the 2D Miniscope (single lens with 2 μm blur). The simulation is performed at 3 light levels (100, 1000, and 10,000 photocounts) using a shift-invariant model with Poisson and read noise added. We use our reconstruction algorithm with an optimized τ value and display the results in Fig. S6. For a fair comparison, we show both the 2D Miniscope raw image and one reconstructed from an image deconvolution process. Our Miniscope3D system has better PNSR than the unprocessed 2D Miniscope data, but the deconvolved 2D Miniscope result

performs the best, as expected. This is because our algorithm is denoising and deblurring. For a scene that does not fit our denoising priors, the processed results would perform worse. Also, note that the loss of PSNR in our system for 2D imaging is a neceessary sacrifice for gaining single-shot 3D imaging capability.

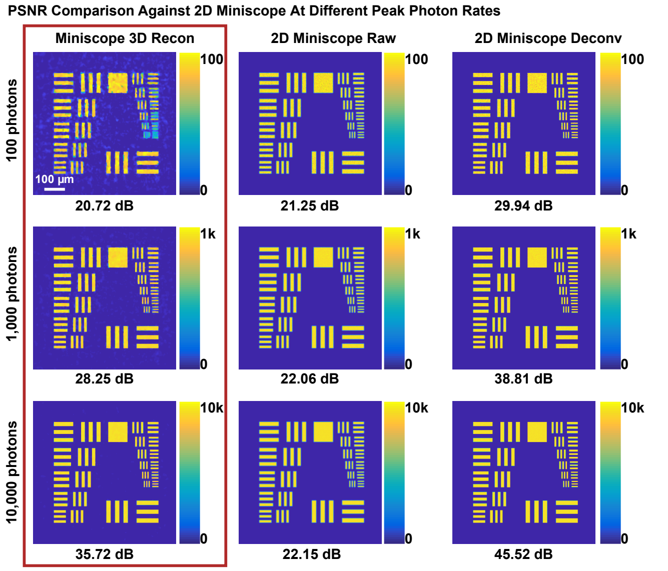

Fig. S6. PSNR comparison of Miniscope3D and 2D Miniscope. (Left) Simulated reconstructions from our system at different light levels. (Middle) 2D Miniscope (simulated) raw measurement. (Right) 2D Miniscope deconvolved reconstructions. The multiplexing properties of our system that enable 3D capabilities result in a loss of PSNR.

8. SPARSITY COMPARISON

Our approach assumes the object to have a sparse representation in some domain. In this paper, we use a general TV sparsity prior to promote gradient sparsity. This is a commonly-used prior for fluorescent imaging for a number of reasons: (1) fluorescent samples are generally sparsely labeled. (2) Even if a 2D slice of the sample is not spatially sparse, it will be sparse when considered with respect to our full 3D volume. (3) If native sparsity does not hold, images are generally sparse in gradient or wavelet domain. (4) Time-priors can further render a volume sparse by only considering temporally-varying information (i.e. neural firings). While it is an NP hard problem to generate a phase transition curve for our system as it requires running a large number of reconstructions of many different classes of objects at each sparsity level, we give an example of how our system performs at different sparsity levels by thresholding a 3D volume to generate different sparsity levels and reporting mean-squared error (MSE) and PSNR. The simulated volume is of a 3D zebrafish dataset. The simulations are done using our 3D shift-varying model and the experimental PSFs from our system. Figure S7 shows MSE and PSNR for the reconstructed volume at different sparsity levels (33%, original volume, to 0.2%, thresholded volume). As expected, our system performs better for sparser volumes. For denser volumes, our system recovers a lower-resolution version of the object and does not fail catastrophically.

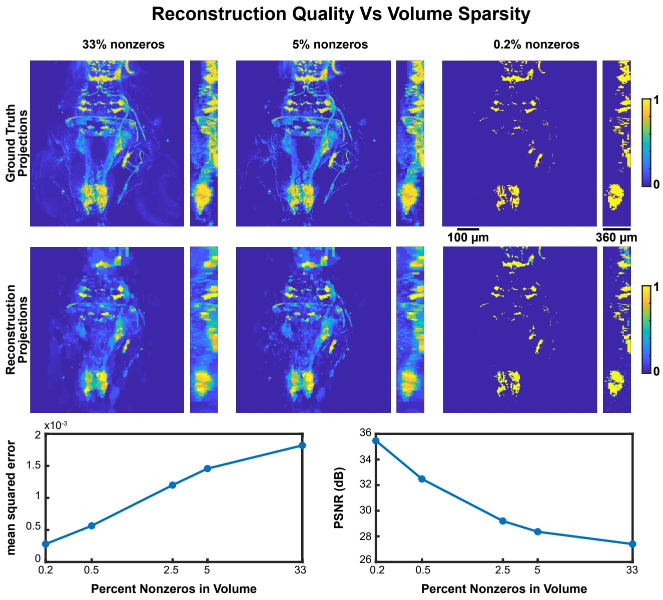

Fig. S7. Simulations of reconstruction quality at different sparsity levels. Maximum intensity projections (y-x, z-x) show the quality of our reconstructions as compared to the ground truth at different sparsity levels. As the volume gets more dense, our reconstruction resolution degrades.

9. GUIDE TO DIFFERENT DESIGNS USING OUR THEORY

Our theory is general and enables other users to design their own optimized 3D microscope targeting different resolutions or volumes-of-interest. To do so, users should implement the following design process:

- For a target lateral resolution, determine the microlens' average clear aperture needed to support that resolution (main paper Sec. *Lateral Resolution*). This also determines the number of microlenses in the phase mask.
- For a target depth range, distribute the focal lengths dioptrically across the depth range.
- Using our optimization criterion, optimize the microlenses positions and aberrations to further enhance the 3D performance.
- Fabricate the phase mask using our adaptive stitching algorithm with a Nanoscribe 3D printer.

10. ADAPTIVE STITCHING

The Nanoscribe 3D printer can only print across a field-of-view (FoV) of 350 μm, and so the 1.8 mm sized phase mask must be printed in multiple stitched blocks, with the mask translating between them. Due to the optical requirements on the microlenses, care needs to be taken when dividing the microlens array into blocks for printing with Nanoscribe. Our adaptive stitching approach aims to print each lens with minimal stitching artifacts. As the clear aperture for each lens is of the same order of magnitude as the maximum printing block size of Nanoscribe, each stitching block will correspond approximately to a single microlens. The center location of each microlens is known, so the problem reduces to dividing the plane in a number of regions, with each region attributed to one of the microlens centres. Preferably, the stitching lines should then fall in the overlapping

region of two (or more) microlenses. We assume that such a division will result in the best possible optical quality.

This problem definition is quite similar to the basic Voronoi segmentation, where we are given a set of points in a plane and the task is to attribute each location in the plane to one of the given points. That problem is solved as follows. For each location in the plane, the distance to all centres is calculated. Attribution to one centre is then decided by it being the closest one (minimum search). As a result, a dividing line is defined by the fact that the distance to two or more centres is equal. The question now is, how can this be adapted to take into account finite shapes?

Rephrasing, we need to define a smooth function in the plane for each microlens followed by attributing locations to microlenses based on a (minimum) search over these different functions. To this end, we will use the height function for each microlens individually and then do a maximum search for the attribution. As a result, segmentation lines would fall exactly at those locations where the height of two or more microlenses are equal (see Fig.6 of main-paper). This is precisely what we want to achieve.

The resulting height-based segmentation is shown in Fig. S8. Here, different slices are shown (50 to 53 μm height). Colored regions need to be printed by Nanoscribe as a single FoV. The different colors correspond to different stitching blocks.

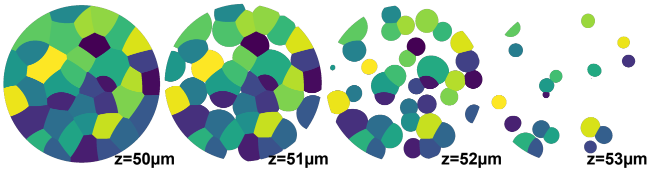

Fig. S8. Different slices are shown, with different colors corresponding to different stitching blocks.

11. RECONSTRUCTED VIDEOS

- **Video 1:** reconstruction of GFP-tagged neurons in 300 μm thick optically cleared mouse brain slice demonstrating single neuron resolution and clearly resolved dendrites running across the volume axially.

- **Video 2:** 3D reconstruction of freely swimming tardigrades. (Left) Raw Data. (Right) Reconstruction of freely moving SYBR-green stained tardigrades.

- **Video 3:** 3D reconstruction of freely swimming tardigrades. (Left) Raw Data. (Right) Reconstruction of freely moving SYBR-green stained tardigrades.

- **Video 4:** 3D reconstruction of freely swimming tardigrades. (Left) Raw Data. (Right) Reconstruction of freely moving SYBR-green stained tardigrades.

- **Video 5:** 3D reconstruction of freely swimming tardigrades. (Left) Raw Data. (Right) Reconstruction of freely moving SYBR-green stained tardigrades.

- **Video 6:** 3D reconstruction of freely swimming tardigrades. (Left) Raw Data. (Right) Reconstruction of freely moving SYBR-green stained tardigrades.

REFERENCES

1. Antipa, N. *et al.* DiffuserCam: lensless single-exposure 3D imaging. *Optica* **5,** 1–9 (2018).
